# Linker Unit Modulation of Polymer Acceptors Enables Highly Efficient Air‐Processed All‐Polymer Solar Cells

**DOI:** 10.1002/advs.202202223

**Published:** 2022-07-10

**Authors:** Ha Kyung Kim, Han Yu, Mingao Pan, Xiaoyu Shi, Heng Zhao, Zhenyu Qi, Wei Liu, Wei Ma, He Yan, Shangshang Chen

**Affiliations:** ^1^ State Key Laboratory of Coordination Chemistry MOE Key Laboratory of High‐Performance Polymer Materials & Technology School of Chemistry and Chemical Engineering Nanjing University Nanjing Jiangsu 210023 P. R. China; ^2^ Department of Chemistry and Hong Kong Branch of Chinese National Engineering Research Center for Tissue Restoration and Reconstruction Hong Kong University of Science and Technology Clear Water Bay Kowloon Hong Kong 999077 P. R. China; ^3^ Hong Kong University of Science and Technology‐Shenzhen Research Institute No.9 Yuexing 1st RD, Hi‐tech Park, Nanshan Shenzhen 518057 P. R. China; ^4^ State Key Laboratory for Super Mechanical Behavior of Materials Xi'an Jiaotong University Xi'an Shaanxi Province 710049 P. R. China; ^5^ Institute of Polymer Optoelectronic Materials and Devices State Key Laboratory of Luminescent Materials and Devices South China University of Technology Guangzhou 510640 P. R. China; ^6^ eFlexPV Limited (Foshan) Guicheng Street Nanhai District Foshan 528200 P. R. China

**Keywords:** air‐processed, all‐polymer solar cells, linker units, polymer acceptors, power conversion efficiencies

## Abstract

A group of regioregular polymer acceptors is synthesized by polymerizing Y6 moieties with different linker units including thiophene, vinylene, 2,2’‐bithiophene, and thieno[3,2‐b]thiophene, and their optoelectrical properties and photovoltaic performances are studied systematically. It is found that the linker units have significant impacts on the backbone planarity, conjugation, and hence optoelectrical properties of polymer acceptors. The vinylene‐based PYF‐V‐*o* polymer shows a smaller dihedral angle between the end groups and vinylene units and a more rigid polymer backbone, thus affording bathochromic absorption and better electron‐transporting capacity. As a result, the PM6:PYF‐V‐*o* based all‐polymer solar cells (all‐PSCs) are able to achieve the highest power conversion efficiency of 16.4% with an unprecedented small voltage loss of 0.49 V. Moreover, the PM6:PYF‐V‐*o* blend exhibits good resistance to environmental stressors and the air‐processed PM6:PYF‐V‐*o* cells can still maintain a high efficiency of 16.1%, which is the best air‐processed all‐PSC efficiency reported to date. This study provides the structural‐property guidance that can be used to facilitate the development of polymer acceptors for all‐PSCs.

## Graphical abstract

The effects of linker units on polymer acceptor properties and device performance are studied, and it shows the linker units have significant impacts on the optoelectrical properties by impacting polymer rigidity and conjugation. The vinylene‐based PYF‐V‐*o* polymer acceptor exhibits a more planar polymer backbone, and its all‐polymer solar cells realize the highest power conversion efficiency of 16.4% and 16.1% when processed in N_2_ and air atmosphere, respectively.

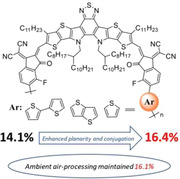

## 1 Introduction

During the past two decades, organic solar cells (OSCs) have drawn extensive research attention due to their outstanding advantages over inorganic counterparts including low cost, solution processibility, low toxicity, and color tunability.^[^ ^1^, ^2^, ^3^, ^4^ ^]^ As a result of intensive research efforts on molecular structures, interface materials, and device optimization, bulk‐heterojunction OSCs with polymer donors and small molecule acceptors (SMAs) are able to reach a power conversion efficiency (PCE) of 19% lately.^[^ ^5^, ^6^, ^7^, ^8^, ^9^, ^10^ ^]^ On the contrary, all‐polymer solar cells (all‐PSCs), with both polymeric donors and acceptors as photoactive materials, have yet reached a similar level of PCEs despite their advantages over SMA‐based devices in terms of morphological stability, mechanical flexibility, and donor‐acceptor compatibility, that can potentially lead to more stable OSCs.^[^ ^11^, ^12^, ^13^ ^]^

**Figure 1:**
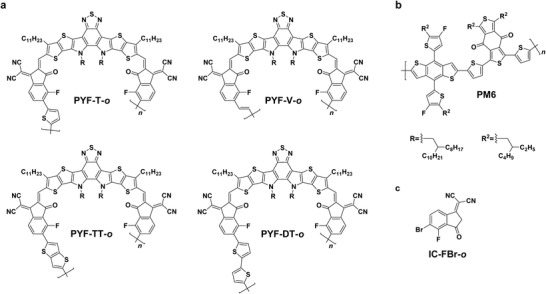
Molecular structures of a) the polymer acceptors, b) PM6, and c) IC‐FBr‐o end group.

Conventional building blocks for polymer acceptors include naphthalene diimide (NDI),^[^ ^14^, ^15^, ^16^, ^17^ ^]^ perylene diimide,^[^ ^18^, ^19^, ^20^ ^]^ B←N heterocycles,^[^ ^21^, ^22^, ^23^ ^]^ and bithiophene imides.^[^ ^24^ ^]^ The resulting polymers, however, typically suffered from several intrinsic weaknesses, such as excessive crystallinity, narrow absorption ranges, and unmatching energy levels. To address these issues, Li et al. proposed a new strategy of embedding SMA units into a polymer's backbone, and they reported a polymer acceptor named PZ1 by polymerizing IDIC SMA with a thiophene linker.^[^ ^25^ ^]^ This strategy enables the resulting polymer acceptors to retain the merits of the original SMAs, i.e., readily tunable structures, high absorption coefficient, suitable bandgap, and broad absorption, which compensate for the aforementioned problems of conventional polymer acceptors. Following the rapid development of Y6‐type SMAs, an effort has been made to incorporate Y6 moiety into polymers.^[^ ^26^, ^27^, ^28^, ^29^, ^30^, ^31^, ^32^, ^33^, ^34^, ^35^ ^]^ In 2020, Min et al. reported a Y6‐based polymer acceptor named PYT that achieved a PCE of 13%, which indeed proved that SMA‐polymerization is a promising strategy to develop Y6‐type polymer acceptors. Nevertheless, one of the drawbacks of previous Y6‐based polymer acceptors is that the bonding position of linker units to end groups of SMAs is not regiospecific. It has already been demonstrated explicitly that polymers’ regioregularity does not only impact morphological properties but also batch‐to‐batch reproducibility, where irregular polymers may end up with inconsistent results.^[^ ^1^, ^35^, ^36^, ^37^ ^]^ As a consequence, a recently designed polymer, PYF‐T*‐o* (**Figure** **1**), adopted a regiospecific fluorinated end group that yielded a high PCE of 15.2%.^[^ ^31^ ^]^ By inducing the *π*‐bridge to a specific position, it was able to form ordered interchain packing and suitable phase separation. However, despite a number of linker units other than thiophene can behave in a similar manner while maintaining the original advantages, not much of the investigation has been made yet and their impacts on polymer properties remain unclear. Besides, most reported all‐PSCs were sensitive to O_2_ or moisture and had to be made in an inert gas‐filled glovebox,^[^ ^39^, ^40^ ^]^ which is not compatible with the scalable manufacturing of organic solar modules. It is urgent to develop high‐performance all‐PSCs that can be fabricated in ambient conditions.

In this work, four regioregular polymer acceptors with different linker units, thiophene (T), vinylene (V), thieno[3,2‐b]thiophene (TT), and 2,2’‐bithiophene (DT), were synthesized and characterized systematically. Our study found that the modulation on the linker units introduced nonnegligible shifts to polymers’ absorption spectra, energy levels, and charge‐transporting properties, which lead to variations in solar cell performance consequently. The vinylene linker unit shows a much smaller dihedral angle (2.6°) with the end group than the other linkers (≈11°), thus affording the resulting PYF‐V‐*o* a more planar and rigid polymer backbone. Meanwhile, the redshifted absorption and high electron mobility of PYF‐V‐*o* facilitate its light‐harvesting and charge transport properties, respectively. As a result, the PM6:PYF‐V*‐o* based all‐PSCs showed the highest PCE of 16.4% with an ultralow voltage loss of 0.49 V. Furthermore, the PM6:PYF‐V‐*o* blend indeed shows weak sensitivity to air and moisture due to the relatively deep energy levels of PYF‐V‐*o*, and the air‐processed PM6:PYF‐V‐*o* all‐PSCs retain a high PCE of 16.1%, which is the record efficiency for air‐processed all‐PSCs reported to date. This study elucidates a relationship between the structures of the linker units and the optoelectrical properties of the polymer acceptors, and guides the development of high‐performance polymer acceptors.

## 2 Results and Discussions

The polymer acceptors in this study were synthesized via Stille coupling reaction between Y‐OD‐FBr and corresponding organic tin reagents (Scheme S1, Supporting Information). A regiospecific IC‐FBr‐*o* end group (Figure 1c) was employed to ensure all resulting polymers are regioregular. Detailed synthetic procedures are described in the Supporting Information. All polymer acceptors are soluble in common organic solvents, including chloroform, toluene, and chlorobenzene. From the thermogravimetric analysis results, PYF‐V*‐o*, PYF‐TT*‐o*, and PYF‐DT*‐o* showed relatively high thermal decomposition temperatures (*T*_d_, 5% weight loss) of 358, 358, and 364 ^⁰^C, respectively (Figure S1, Supporting Information), which are all comparable to the reported *T*_d_ of PYF‐T*‐o*. Therefore, the linker units have negligible impacts on the thermal stability of the polymer acceptors.

**Figure 2:**
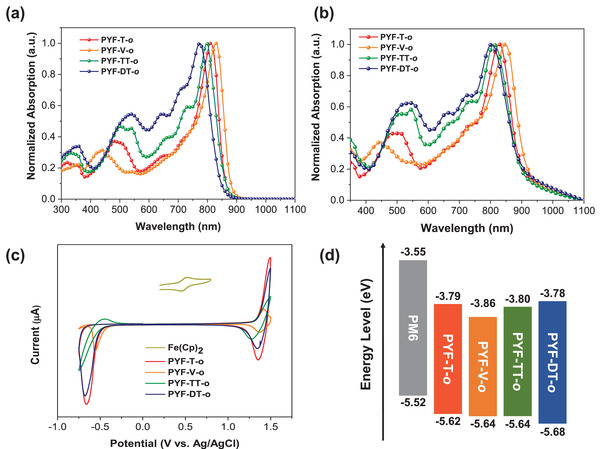
a) Normalized UV‐vis absorption spectra of the polymer acceptors in dilute solution state (1.0 × 10^–5^ M). b) Normalized UV‐vis spectra of the polymer acceptors in thin film state. c) Cyclic voltammetry curves of the polymer acceptors. d) Energy levels of the PM6 and four polymer acceptors.

UV‐vis absorption spectra of the polymer acceptors in both dilute solution and thin film states were obtained as shown in **Figure** **2** and Figure S2, Supporting Information. When dissolved in chloroform, the absorption maxima (*λ*_max,sol_) of PYF‐T‐*o*, PYF‐V‐*o*, PYF‐TT‐*o*, and PYF‐DT‐*o* were located at 812, 830, 798, and 773 nm, respectively (**Table** **1**). The bathochromic shift observed in PYF‐V‐*o* is a strong indication of the improved intramolecular conjugation facilitated by the vinylene units, as a result of more planar polymer backbone of PYF‐V‐*o*. Similar trend was also observed in the thin film state, and PYF‐V‐*o* gave the highest absorption maximum (*λ*_max,film_) at 847 nm, followed by PYF‐T‐*o* (829 nm), PYF‐TT‐*o* (811 nm), and PYF‐DT‐*o* (803 nm). The *λ*_max_ in both film and solution states tend to blueshift as the sizes of the linker units increase. From this point of view, it can be estimated that the conjugation over the entire polymer backbone can be enhanced with a smaller and more rigid linker unit. In contrast, a shorter wavelength absorption ranging from 400 to 600 nm is enhanced and redshifted as the sizes of the linkers grow. It can be attributed to a larger conjugation length of the linker unit itself, while it has minor impacts on the overall conjugation of the polymer backbone. The same phenomenon can be observed from the 0–1 and 0–2 absorptions.

**Table 1:** Optical and electrochemical properties of the polymer acceptors

Polymer acceptor	*λ*_max,sol_ [nm]	*λ*_max,film_ [nm]	*λ*_onset,film_ [nm]	* E *_g_ ^a)^ [eV]	HOMO^b)^ [eV]	LUMO^b)^ [eV]
PYF‐T*‐o*	812	829	893	1.39	−5.62	−3.79
PYF‐V*‐o*	830	847	908	1.37	−5.64	−3.86
PYF‐TT*‐o*	798	811	891	1.39	−5.64	−3.80
PYF‐DT*‐o*	773	803	887	1.40	−5.68	−3.78

^a)^ Calculated from the absorption onset of the films

^b)^ Estimated from the onsets of the CV curves.

To further verify the effects of the linker units on the conjugation of the polymer acceptor, density functional theory (DFT) calculations were carried out at the B3LYP/6‐31G (*d, p*) level. The dihedral angles between the end groups and the linker units are of particular interest to evaluate the planarity of the polymer backbones. The average dihedral angle between the end group and the vinylene unit for PYF‐V*‐o* is much smaller (≈2^o^) than the other three polymers (≈11^o^) (**Table** **2**). Furthermore, the average distance between the fluorine atom on the end group and the hydrogen atom on the vinylene linker was calculated to be 2.15 Å, smaller than the sum of Van der Waals radii of the two atoms. This is indicative of the non‐covalent interaction between them, resulting a pseudo‐six‐membered ring structure. Such a planar structure enhances the conjugation along the PYF‐V‐*o* backbone, which is consistent with the results we observed in UV‐vis absorption spectra (Figure 2b). As a result, the visualized lowest unoccupied molecular orbital (LUMO) level of PYF‐V*‐o* (Figure S3, Supporting Information) shows the electron density is delocalizing over the entire linker unit. Contrastingly, not much of the delocalized electrons are observed on the linker moieties of the other three polymers. Subsequently, energy levels of the polymer acceptors were estimated using cyclic voltammetry with Fc/Fc^+^ as an external standard (Figure 2, and Table 1). Compared to PYF‐T*‐o* (−5.62/−3.79 eV), PYF‐V*‐o* showed a downshifted LUMO level (−3.86 eV), which indicates a weaker electron‐donating effect of the vinylene linker compared to the other aromatic rings. Such a deep LUMO energy level is beneficial to make PYF‐V‐*o* less vulnerable to oxidation caused by ambient species. Also, PYF‐DT*‐o* showed a downshifted highest occupied molecular orbital (HOMO) level (−5.68 eV) that is attributed to a larger bandgap as the *λ*_max_ blueshifts. The results are in a similar trend to the DFT calculated values.

**Table 2:** Average dihedral angles between the end group and linker units of the polymer acceptors

Polymer acceptor	Angle [^o^]
PYF‐T*‐o*	12.87
PYF‐V*‐o*	2.64
PYF‐TT*‐o*	11.64
PYF‐DT*‐o*	10.23

**Figure 3:**
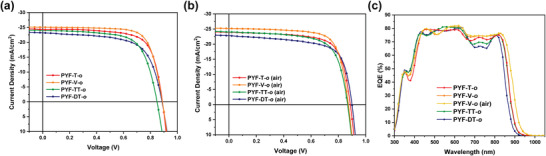
*J–V* characteristic curves of the devices fabricated under a) N_2_ and b) ambient air, and c) EQE spectra of the all‐PSCs.

Photovoltaic performance of each polymer acceptor was compared in a conventional device structure of glass/ITO/PEDOT:PSS/PM6:polymer acceptor/PNDIT‐F3N/Ag fabricated inside a N_2_‐filled glovebox. The results are summarized in **Table** **3** and Table S1, Supporting Information, and the *J–V* curves are plotted in **Figure** **3**a. The reference PYF‐T*‐o*‐based devices performed consistently as previously reported, yielding a PCE of 15.4%. PYF‐V*‐o*‐based devices yielded a comparable open‐circuit voltage (*V*_OC_) of 0.884 V yet improved short‐circuit current density (*J*_SC_) and fill factor (FF) of 25.1 mA cm^–2^ and 73.7% respectively, which lead to a higher PCE of 16.4%. Such a PCE is among the highest efficiencies reported for all‐PSCs. On the other hand, both PYF‐TT*‐o* and PYF‐DT*‐o*‐based devices showed lower *J*_SC_ and FF, resulting in inferior PCEs of 14.6% and 14.1% respectively. In addition to the devices fabricated in N_2_ filled glovebox, we also made the all‐PSCs at ambient conditions (50% RH and room temperature), and the resultant device photovoltaic performance are shown in Figure 3b and **Table** **4**. Encouragingly, air‐processed PM6:PYF‐V‐*o* all‐PSCs still maintained an impressive PCE of 16.1%. To our best knowledge, this is the best PCE reported for air‐processed all‐PSCs (Table S2, Supporting Information). Such a small efficiency derate between N_2_ atmosphere fabricated and air‐processed cells can be attributed to the relatively lower LUMO levels of PYF‐V‐*o* due to both end group fluorination effects and more planar vinylene units, which make electron charge carriers more resistant to the ambient oxidation energetically during the device fabrication in air. In order to further investigate the disparity in their *J*_SC_s, external quantum efficiency (EQE) spectra of four systems were obtained in Figure 3c. The integrated *J*_SC_s calculated based on the spectra agree well with those derived from *J–V* characteristics. An obvious difference was observed in the region between 670 ≈ 820 nm, where the polymer acceptors are mainly in charge. The PYF‐V*‐o*‐based devices achieved the highest photon response reaching ≈75%, contributing to the enhanced photocurrent generation. In contrast, PYF‐DT*‐o*‐based blends could barely approach 65%. The decreasing trend in EQE perfectly corresponds to the increasing sizes of the linker units, proving the rigidity of the linker unit facilitates the charge transport. It is also worthwhile mentioning that the PYF‐V‐*o*‐based all‐PSCs delivered the smallest voltage loss of 0.49 V (Table S3, Supporting Information). Considering that four polymer acceptors possess comparable optical bandgaps and absorption ranges, the radiative voltage losses of four groups of all‐PSCs are similar. Therefore, the variation in device voltage losses mainly comes from their differences in non‐radiative loss, which are correlated with semiconductor energy disorders. The more rigid polymer backbone of PYF‐V‐*o* is beneficial for reducing the degree of energetic disorders and the nonradiative recombination pathways, which can explain the smallest voltage loss in the PM6:PYF‐V‐*o* based all‐PSCs. Device stabilities were also tested by light‐soaking four types of all‐PSCs under one sun luminescence at open‐circuit conditions for over 300 h. As shown in Figure S4, Supporting Information, four all‐PSCs were able to maintain over 80% of their original PCEs after being illuminated.

**Table 3:** Device performance of the all‐PSCs based on PM6:polymer acceptor

Polymer acceptor	*V*_OC_ [V]	*J*_SC_ [mA cm^–2^]	*J*_cal_ [mA cm^–2^]	FF [%]	PCE [%]
PYF‐T*‐o*	0.889	24.3	23.7	71.5	15.4
PYF‐V*‐o*	0.884	25.1	24.9	73.7	16.4
PYF‐TT*‐o*	0.869	24.1	23.6	69.9	14.6
PYF‐DT*‐o*	0.899	23.2	22.4	67.7	14.1

**Table 4:** Device performance of the all‐PSCs based on PM6:polymer acceptor fabricated at ambient conditions

Polymer acceptor	*V*_OC_ [V]	*J*_SC_ [mA cm^–2^]	FF [%]	PCE [%]
PYF‐T*‐o* (air)	0.887	23.9	70.8	15.0
PYF‐V*‐o* (air)	0.874	25.2	72.9	16.1
PYF‐TT*‐o* (air)	0.867	24.0	68.5	14.3
PYF‐DT*‐o* (air)	0.899	22.8	67.8	13.9

**Figure 4:**
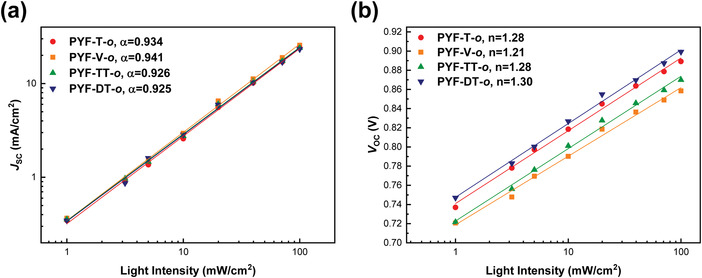
Light‐intensity‐dependent a) *J*_SC_ and b) *V*_OC_ curves of the all‐PSCs. Solid lines are fitting curves.

The dependence of *J–V* characteristics on light intensity (*P*) can provide useful information on the recombination loss of devices and has been widely used to describe monomolecular and/or bimolecular recombination. To further investigate the charge recombination behaviors of the four groups of devices, the incident light intensity dependence of the *J*_SC_ was first plotted with a fitting formula of *J*_SC_∝*P^*α*^*. According to **Figure** **4**, the *α* values were fitted to be 0.934, 0.941, 0.926, and 0.925 for PM6:PYF‐T*‐o*, PM6:PYF‐V*‐o*, PM6:PYF‐TT*‐o*, and PM6:PYF‐DT*‐o* respectively, implying the least bimolecular recombination of the PYF‐V*‐o* based blend. The *V*_OC_ was also plotted against log*P* with the slope of *nk_B_T/q*, where *n* is the ideal factor, *k_B_* is the Boltzmann constant, *T* is the temperature, and *q* is the elementary charge. The PM6:PYF‐V*‐o* blend afforded a smaller *n* value of 1.21 than PYF‐T*‐o* (1.28), PYF‐TT*‐o* (1.28), and PYF‐DT*‐o* (1.30) based devices, respectively, indicating the highest suppression of trap‐assisted Shockley‐Read‐Hall (SRH) recombination inside the PM6:PYF‐V*‐o* based devices. All these results affirm that the recombination loss in the PM6:PYF‐V‐*o* system has been significantly mitigated, which can partially explain its smallest voltage loss and higher PCE than the other cases.

In order to understand the improved FF of the PM6:PYF‐V‐*o*, both electron (*μ_e_*) and hole (*μ_h_*) mobilities of the four all‐PSCs were characterized with the space‐charge‐limit current method (Figure S5 and Table S4, Supporting Information). *μ_e_* and *μ_h_* of the PM6:PYF‐V*‐o* blend were measured to be 8.6 × 10^–4^ and 6.9 × 10^–4^ cm^2^ V^–1^ s^–1^ respectively, both of which exceeded those of the PM6:PYF‐T*‐o* blend (*μ_e_* = 7.8 × 10^–4^ cm^2^ V^–1^ s^–1^ and *μ_h_* = 6.6 × 10^–4^ cm^2^ V^–1^ s^–1^), contributing to a higher FF in the PYF‐V‐*o* based all‐PSCs. In contrast, PM6:PYF‐TT*‐o* (*μ_e_* = 7.2 × 10^–4^ cm^2^ V^–1^ s^–1^ and *μ_h_* = 6.5 × 10^–4^ cm^2^ V^–1^ s^–1^) and PM6:PYF‐DT*‐o* (*μ_e_* = 6.8 × 10^–4^ cm^2^ V^–1^ s^–1^ and *μ_h_* = 5.6 × 10^–4^ cm^2^ V^–1^ s^–1^) blends displayed inferior charge mobilities. Notably, the charge mobilities tend to increase as the dihedral angles between the end group and the linker unit decrease. This indicates a positive influence of the rigidity of the linkers on the charge transport due to the enhanced conjugation along the polymer backbone.

**Figure 5:**
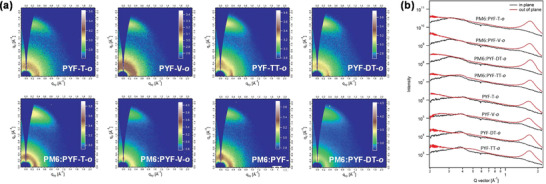
a) 2D GIWAXS patterns and b) the corresponding 1D GIWAXS line‐cuts in IP and OOP directions of PYF‐T‐*o*, PYF‐V‐*o*, PYF‐TT‐*o*, and PYF‐DT‐*o* neat and blend films.

Morphology is critical in determining solar cell performance, and the effects of the linker units on film morphology are worthy of investigation naturally. Grazing incidence wide‐angle X‐ray scattering (GIWAXS) measurements were carried out for both pristine and blend films in order to investigate their molecular packing and crystallinities. The 2D GIWAXS patterns are displayed in **Figure** **5**a, and the corresponding in‐plane (IP) and out‐of‐plane (OOP) line cuts are depicted in Figure 5b. All films exhibit intense (010) diffraction peaks in the OOP direction, indicating predominant adoption of “face‐on” orientations that is beneficial to charge transport in the vertical direction across the electrodes. The (010) peaks for four pristine films are all located at ≈1.65 Å^–1^, which corresponds to a comparable *π*–*π* stacking distance (*d*_*π*‐*π*_) of 3.8 Å. PYF‐TT‐*o* and PYF‐DT‐*o* show slightly longer coherence length (29 Å and 27 Å) compared with those of PYF‐T‐*o* and PYF‐V‐*o* (23 Å and 24 Å) probably due to twisted conformations of polymer backbones. When blended with PM6, the crystallinity features of the four blends are quite similar with the (010) diffraction peaks located at ≈1.68 Å, which should be contributed by both PM6 and the polymer acceptors but it is hard to distinguish from each other. The surface topography of four blend films characterized by atomic force microscopy also shows minor differences among the four blend films (Figure S6, Supporting Information), except the PM6:PYF‐V‐*o* blend exhibited slightly higher root mean square (RMS) roughness. It is likely to be attributed to the most rigid polymer backbone of the PYF‐V‐*o*, that inducing a stronger aggregation when blended into devices, giving a rougher surface than the rest of the samples.^[^ ^41^, ^42^ ^]^ Higher RMS roughness may also contribute to a better charge extraction due to an increased contact area with the electrodes.^[^ ^43^, ^44^ ^]^

## 3 Conclusion

In conclusion, we report four polymer acceptors consisting of alternating Y6 moiety and different linker units including thiophene, vinylene, 2,2’‐bithiophene, and thieno[3,2‐b]thiophene. The vinylene‐based PYF‐V‐*o* showed a much smaller dihedral angle and a more rigid polymer backbone compared to the other three polymer acceptors, resulting in a bathochromic shift in its UV‐vis absorption spectrum. This also enhances the overall conjugation of the polymer that strengthens the intramolecular charge transfer. Normally structured devices were fabricated and the air‐processed PM6:PYF‐V‐*o* all‐PSCs achieved the highest PCE of 16.1% with a small voltage loss of <0.50 V. Such an efficiency is the highest PCEs for the air‐processed all‐PSCs reported so far. The work elucidates the correlations between the linker units and the properties of the polymer acceptors, and provides a feasible option in manipulating polymer structures for more efficient all‐PSCs.

## Conflict of Interest

The authors declare no conflict of interest.

## Supporting information

Supporting Information

## Data Availability

The data that support the findings of this study are available from the corresponding author upon reasonable request.
