# Supplementary material for: Linker Unit Modulation of Polymer Acceptors Enables Highly Efficient Air‐Processed All‐Polymer Solar Cells
Source: Adv Sci (Weinh). 2022 Jul 10;9(25):2202223. doi: 10.1002/advs.202202223 (PMC9443469; doi:10.1002/advs.202202223)
Supplement: Supplementary file 1 — Supporting Information [file ADVS-9-2202223-s001.pdf]

ADVANCED SCIENCE
Open Access

Supporting Information

for *Adv. Sci.*, DOI 10.1002/advs.202202223

Linker Unit Modulation of Polymer Acceptors Enables Highly Efficient Air-Processed All-Polymer Solar Cells

Ha Kyung Kim, Han Yu, Mingao Pan, Xiaoyu Shi, Heng Zhao, Zhenyu Qi, Wei Liu, Wei Ma, He Yan* and Shangshang Chen**

Supporting Information

Linker Unit Modulation of Polymer Acceptors Enables Highly Efficient Air-Processed All-Polymer Solar Cells

*Ha Kyung Kim[1,2], Han Yu[2,3 *], Mingao Pan[2], Xiaoyu Shi[1], Heng Zhao[4], Zhenyu Qi[2], Wei Liu[2], Wei Ma[4], He Yan[2,3,5,6 *], Shangshang Chen[1 *]*

[1] H. K. Kim, X. Shi, Prof. S. Chen
State Key Laboratory of Coordination Chemistry, School of Chemistry and Chemical Engineering, Nanjing University, Nanjing, Jiangsu 210023, China
E-mail: schen@nju.edu.cn

[2] H. K. Kim, Dr. H. Yu, M. Pan, Z. Qi, W. Liu, Prof. H. Yan
Department of Chemistry and Hong Kong Branch of Chinese National Engineering Research Center for Tissue Restoration and Reconstruction, Hong Kong University of Science and Technology, Clear Water Bay, Kowloon, Hong Kong 999077, China.
E-mail: hyuak@connect.ust.hk; hyan@ust.hk

[3] Dr. H. Yu, Prof. H. Yan
Hong Kong University of Science and Technology-Shenzhen Research Institute, No.9 Yuexing 1st RD, Hi-tech Park, Nanshan, Shenzhen 518057, China

[4] H. Zhao, Prof. W. Ma
State Key Laboratory for Super Mechanical Behavior of Materials, Xi'an Jiaotong University, Xi'an, Shaanxi Province, 710049, China.

[5] Prof. H. Yan
Institute of Polymer Optoelectronic Materials and Devices, State Key Laboratory of Luminescent Materials and Devices, South China University of Technology, Guangzhou 510640, China.

[6] Prof. H. Yan
eFlexPV Limited (Foshan), Guicheng Street, Nanhai District, Foshan, 528500, P. R. China.

General Information. ^{1}H NMR spectra were tested on a Bruker AV-400 MHz NMR spectrometer. Chemical shifts were reported in parts per million (ppm, δ). ^{1}H NMR spectra were referenced to tetramethylsilane (0 ppm) for $CDCl_3$.

Materials. Tetrahydrofuran and toluene were freshly distilled with sodium and calcium hydride respectively before use. **Y-OD-FBr** was synthesized according to the previous report [1]. Linker units including 2,5-bis(trimethylstannyl)thiophene, 2,5-bis(trimethylstannyl)thieno[3,2-b]thiophene, 2,2'-bis(trimethylstannyl)-5,5'-bithiophene, and 1,2-di(tributylstannyl)ethene were purchased from chemicals sources and underwent recrystallization before the polymerization reaction.

Molecular Weight Measurement. Molecular weights of the polymers were measured with a high-temperature gel permeation chromatography (HT-GPC, Agilent PL-GPC220) at 160 ^oC with 1,2,4-trichlorobenzene as the eluent and polystyrene as the standard.

TGA Measurements. The TGA thermograms of the materials were tested using the TA instrument Q5000 SA Thermogravimetric Analyzer. Under N_2 atmosphere, the materials were heated from room temperature to 800 ^oC at the heating rate of 10 ^oC min^{-1}. Baseline and temperature were calibrated with sapphire and indium.

Optical characterizations. All UV-vis absorption spectra were acquired on a Perkin Elmer Lambda 20 UV/VIS Spectrophotometer. Film samples were spin-casted on ITO substrates and solution samples were dissolved in chloroform with a concentration of 1.0×10^{-5} M.

Electrochemical characterizations. Cyclic voltammetry was carried out on a CHI610E electrochemical workstation with a three-electrode system consisting of a Ag/AgCl reference

electrode, a Pt counter electrode, and a glassy carbon working electrode. 0.1 mol L^{-1} tetrabutylammonium hexafluorophosphate in anhydrous acetonitrile was used as the supporting electrolyte. The solid films on the working electrode were drop-cased from a chloroform solution of samples in a concentration of 5 mg mL^{-1}. Potentials were referenced to the ferrocenium/ferrocene couple by using ferrocene as an external standard in an acetonitrile solution. The scan rate was 100 mV s^{-1}.

Solar cell fabrication and testing. Devices were fabricated on the indium tin oxide (ITO) patterned glass with a conventional configuration of glass/ITO/PEDOT:PSS/PM6:polymer acceptor/PNDIT-F3N/Ag. A thin layer of PEDOT:PSS (CLEVIOS 4083, ~40 nm) was spin-coated onto the ITO substrate at 4000 rpm for 40 s, and dried at 150 ^{o}C for 15 mins. The substrates were then transferred into a glove box filled with N_2. The PM6:polymer acceptor blend in a ratio of 1:1.2 (w/w) was dissolved in chloroform at a concentration of 16 $mgmL^{-1}$ with 2% (v/v) CN as an additive. The solution was stirred vigorously for 2 hrs at 60 ^{o}C and spin-casted on the ITO/PEDOT:PSS substrates at 4500 rpm for 40 s. The optimal blend thickness measured on a Bruker Dektak XT stylus profilometer was around 100 nm. Subsequently, a thin layer of PNDIT-F3N (~7 nm) was then cast onto the top of active layers and a Ag layer (~100 nm) was deposited inside a thermal evaporator under the reduced pressure (5×10^{-5} Pa). The air-processed all-PSCs were fabricated at 50% RH and room temperature following the same spin-coating conditions as above. The *J-V* curves were measured with a Keithley 2400 Source Meter in air. The photocurrent was measured under AM 1.5G (100 mW cm^{-2}) using a Newport solar simulator in the air. The light intensity was calibrated using a standard Si diode to bring spectral mismatch to unity.

EQE measurements. EQEs were measured using an Enlitech QE-S EQE system equipped with a standard SI diode. Monochromatic light was generated from a Newport 300 W lamp source.

SCLC measurements. The charge mobilities of blend films were measured using the space-charge limited current (SCLC) method. The device structure for electron mobility measurements was ITO/ZnO/blend film/PNDIT-F3N/Al, and for hole mobility measurements it was ITO/PEDOT:PSS/blend film/MoO_3/Al. The mobility can be calculated by fitting the dark current to the model of a single carrier SCLC, $J = 9\varepsilon_0\varepsilon_r\mu V^2/8L^3$, where J is the current density, ε_0 is the permittivity of vacuum, ε_r is the relative dielectric constant of the transport medium component, μ is the charge mobility, and L is the film thickness. V is the difference of applied voltage (V_{app}) and offset voltage (V_{BI}). The mobility of charge carriers can be calculated from the slope of $J^{1/2} \sim V$ curves.

AFM analysis. AFM measurements were performed by using Dimension 3100 Scanning Probe Microscope in tapping mode. The samples were spin-casted on PEDOT:PSS covered ITO substrates.

GIWAXS characterization. Grazing-incidence Wide Angle X-ray Scattering (GIWAXS) measurements were performed at beamline 7.3.3 at the Advanced Light Sources. Samples were prepared on Si substrates using identical blend solutions as those used in device fabrication. The 10 keV X-ray beam was incident at grazing angles ranging from 0.12° to 0.16°, selected to maximize the scattering intensity from the tested samples. The scattered X-ray was detected using a Dectris Pilatus 2M photon-counting detector. The coherence length was calculated using the Scherrer equation, where $CL = 2\pi K/\Delta q$, where K is a shape factor, and Δq is the full-width at half-maximum of the peak.

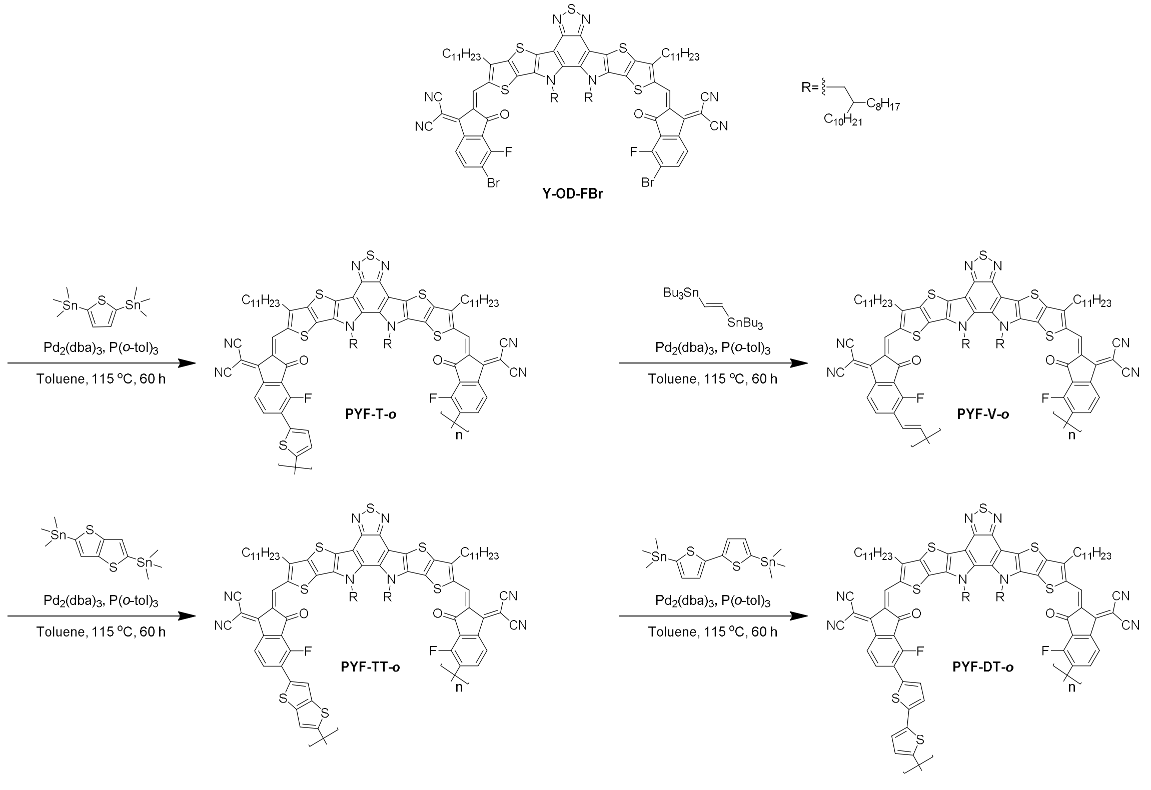

Scheme S1. Synthesis of PYF-T-*o*, PYF-V-*o*, PYF-TT-*o*, and PYF-DT-*o*.

General Polymerization Procedure

To a 10 mL Schlenk tube with a stir bar, **Y-2OD-FBr** (50 mg, 0.0262 mmol), corresponding tin reagent (0.0262 mmol), tris(dibenzylideneacetone)dipalladium(0) (1.44 mg, 1.57×10^{-3} mmol), tri(*o*-tolyl)phosphine (1.99 mg, 6.55×10^{-3} mmol), and toluene (2 mL, 0.013 M) were added. The reaction mixture was stirred at 115 ^{o}C for 60 h. It was cooled down to room temperature and precipitated with methanol. The resulting solid was subjected to Soxhlet extraction with methanol, hexane, acetone, and chloroform. The chloroform extract was precipitated with methanol and then dried under vacuum. The ^{1}H NMR spectra of three new polymers can be found in Figures S6 to S8.

Polymer characterization:

PYF-T-*o*: Yield: 65%. Darker purple. GPC: M_n= 13.4 kDa, M_w= 23.3 kDa, PDI= 1.74.

PYF-V-*o*: Yield: 71%. Dark green. GPC: M_n= 18.4 kDa, M_w= 28.9 kDa, PDI= 1.57.

PYF-TT-*o*: Yield: 74%. Dark purple. GPC: M_n= 25.2 kDa, M_w= 66.8 kDa, PDI= 2.65.

PTF-DT-*o*: Yield: 68%. Dark purple. GPC: M_n= 12.8 kDa, M_w= 20.4 kDa, PDI= 1.60.

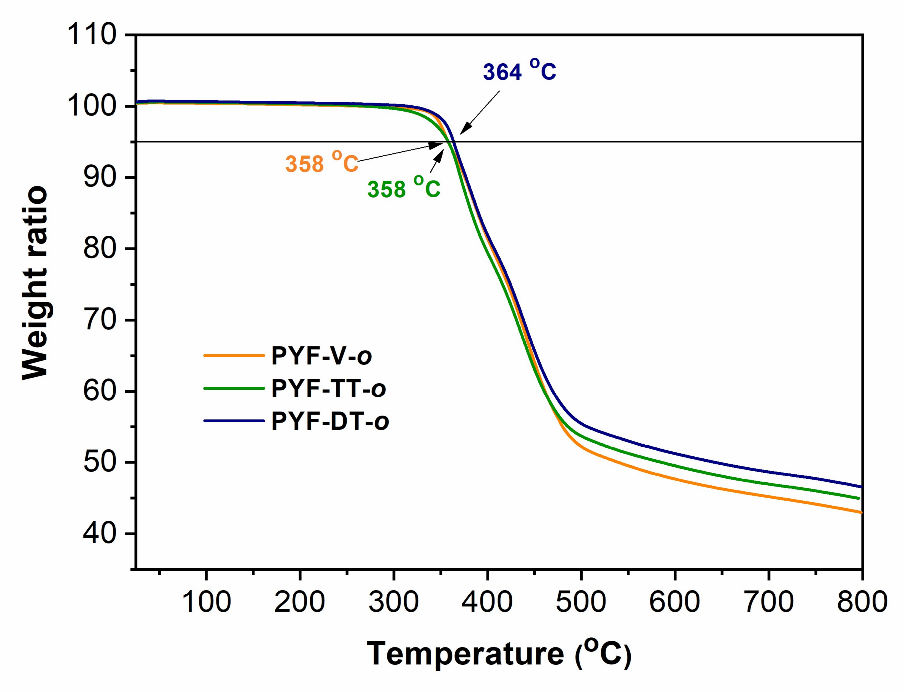

Figure S1. Thermogravimetric analysis curves of PYF-V-*o*, PYF-TT-*o*, and PYF-DT-*o*.

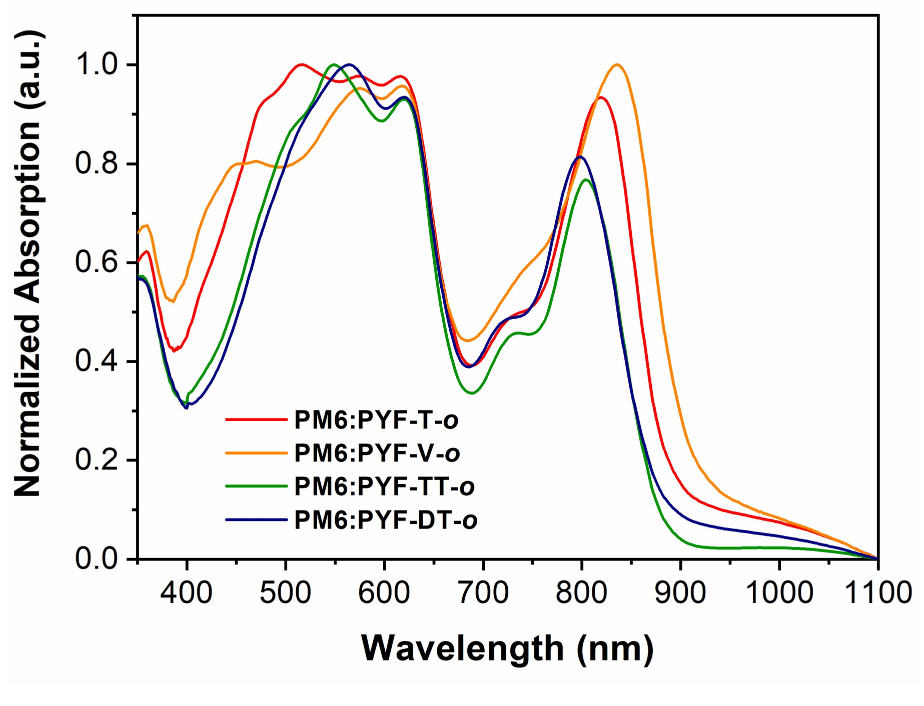

Figure S2. Normalized UV-vis spectra of PM6:polymer acceptor blend films.

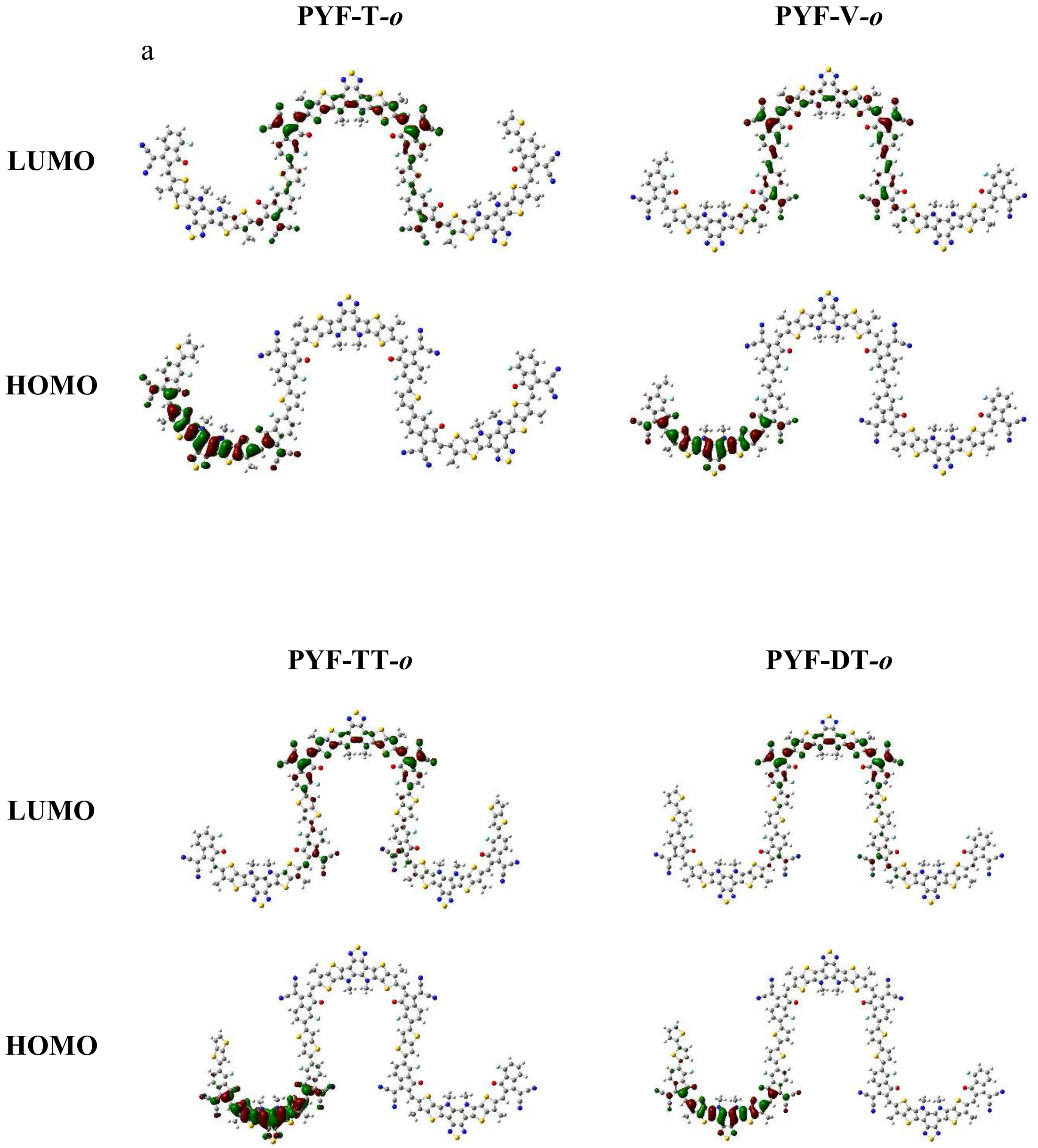

Figure S3. DFT-optimized frontier molecular orbitals of the polymer acceptors.

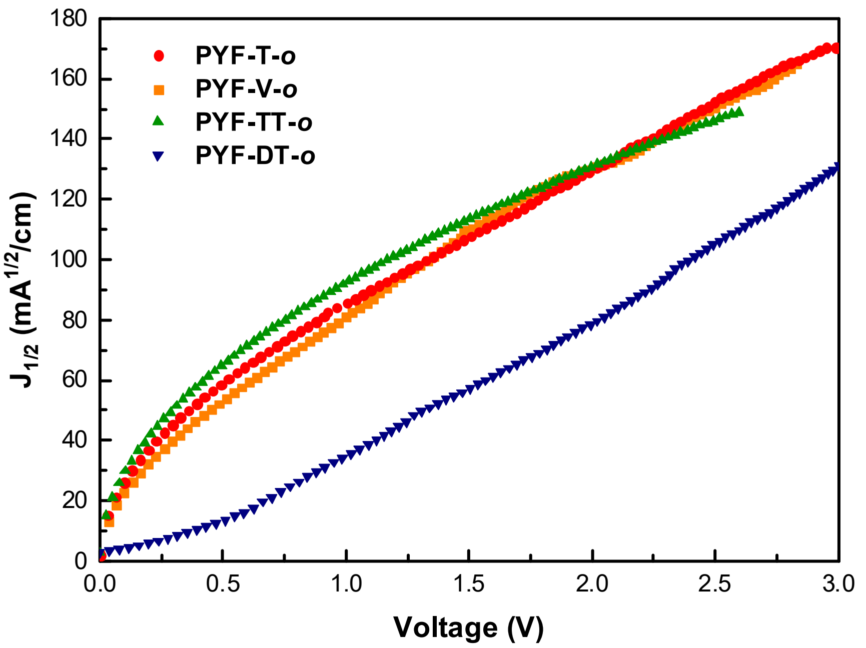

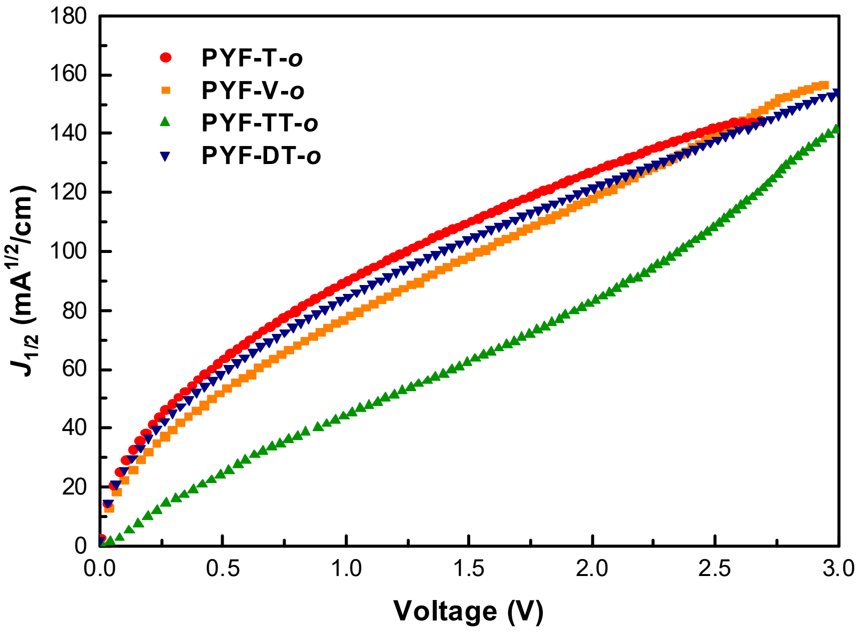

Figure S4. $J^{1/2}$-V characteristic curves of electron-only devices (upper) and hole-only devices (lower).

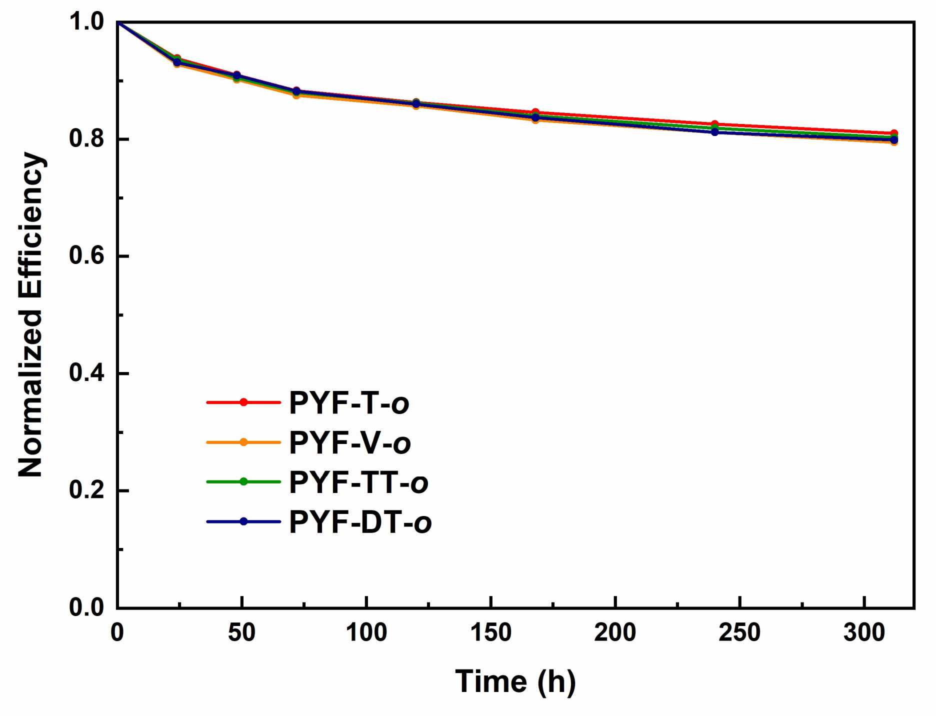

Figure S5. Stability results of the encapsulated devices at open-circuit conditions under 1 sun illumination in air.

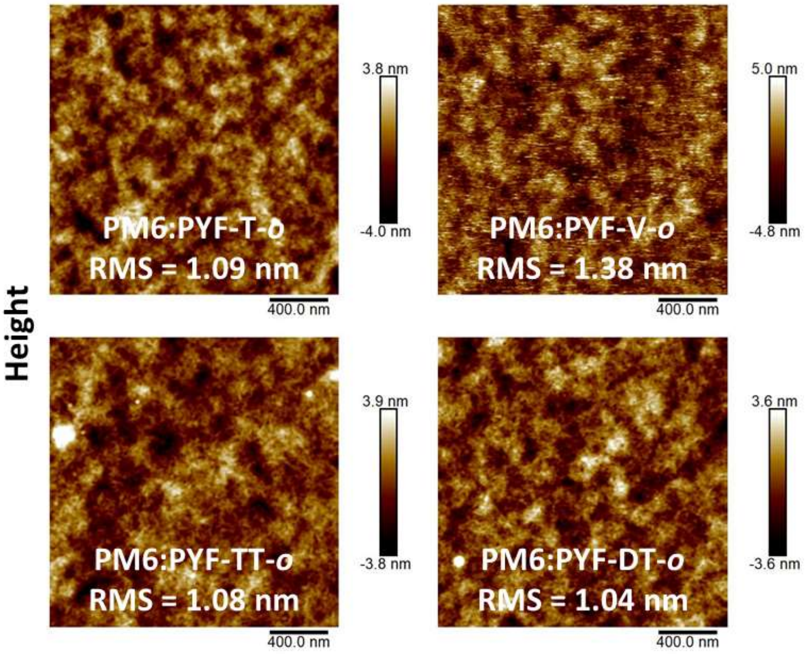

Figure S6. AFM height images of PM6:PYF-T-*o*, PM6:PYF-V-*o*, PM6:PYF-TT-*o*, and PM6:PYF-DT-*o* blend films.

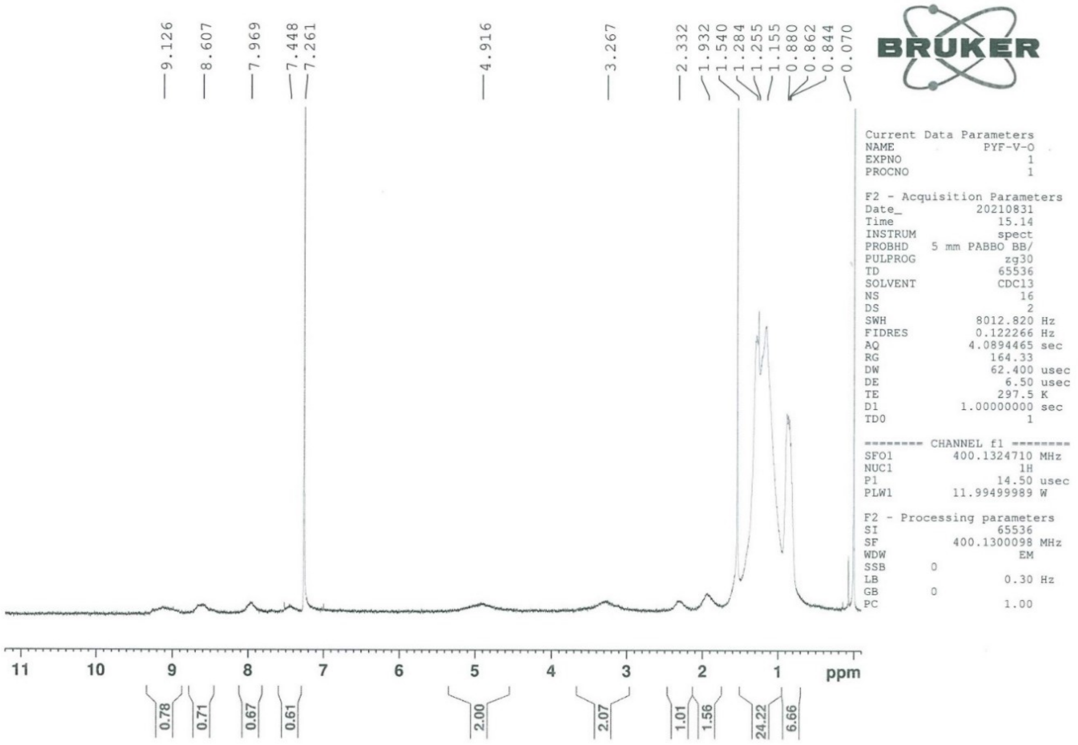

Figure S7. ^{1}H NMR spectrum of PYF-V-*o*.

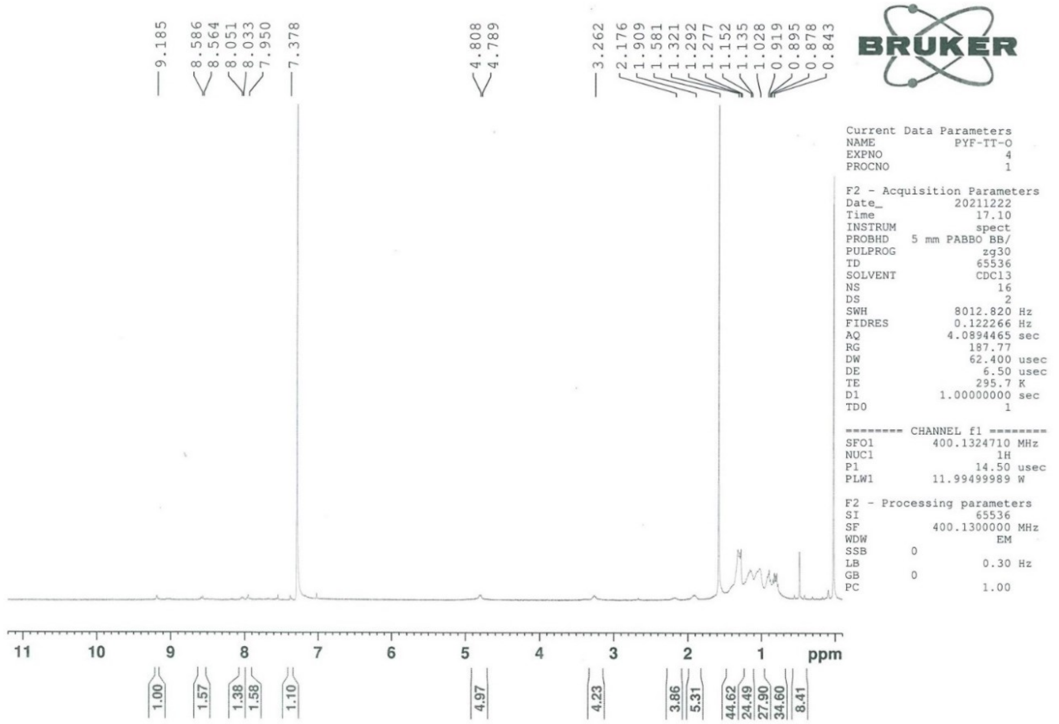

Figure S8. ^{1}H NMR spectrum of PYF-TT-*o*.

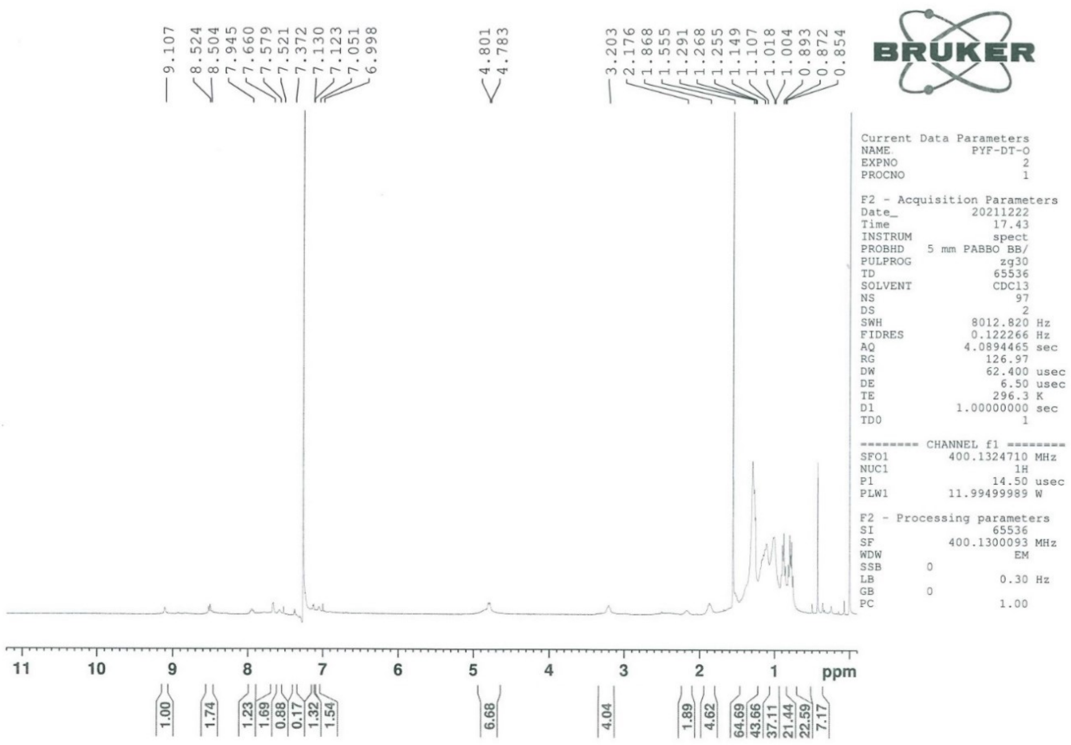

Figure S9. ^{1}H NMR spectrum of PYF-DT-*o*.

Table S1. Average device performance of the all-PSCs based on PM6:polymer acceptor fabricated in N_2-filled glovebox.

Polymer acceptor	V_{OC} [V]	J_{SC} [mA cm^{-2}]	FF [%]	PCE [%]
PYF-T-*o*	0.889 ± 0.001	24.09 ± 0.25	70.8 ± 0.8	15.34 ± 0.11
PYF-V-*o*	0.872 ± 0.004	25.15 ± 0.12	72.3 ± 0.7	15.90 ± 0.23
PYF-TT-*o*	0.867 ± 0.003	23.85 ± 0.23	68.9 ± 1.0	14.25 ± 0.42
PYF-DT-*o*	0.898 ± 0.002	22.98 ± 0.27	67.3 ± 0.5	13.89 ± 0.27

Table S2. Device performance of the air-processed all-PSCs reported recently.

Active Layers	V_{OC} [V]	J_{SC} [mAcm^{-2}]	FF	PCE [%]	Ref.
P3HT:DPP-Pht$_2$	0.89	5.91	0.50	3.3	[2]
PBDT-TS1:PPDIODT	0.74	12.79	0.53	5.2	[3]
PBDB-T:N2200	0.86	11.10	0.65	6.2	[4]
PTB7-Th:PDI-V	0.74	15.30	0.64	7.3	[5]
PTzBI:N2200	0.84	14.86	0.67	8.4	[6]
PM6:PY2F-T	0.86	23.64	0.71	14.1	[7]
PM6:L14	0.95	21.19	0.74	14.9	[8]
PM6:PYF-T-*o*	0.887	23.9	0.708	15.0	This work
PM6:PYF-V-*o*	0.874	25.2	0.729	16.1	This work
PM6:PYF-TT-o	0.867	24.0	0.685	14.3	This work
PM6:PYF-DT-*o*	0.899	22.8	0.678	13.9	This work

Table S3. Energy loss analysis of the all-PSCs based on four polymer acceptors.

Polymer acceptor	E_g (eV)	qV_{OC} (eV)	E_{loss} (eV)	ΔE_1 (eV)	ΔE_2 (eV)	ΔE_3 (eV)
PYF-T-*o*	1.39	0.889	0.50	0.26	0.02	0.22
PYF-V-*o*	1.37	0.884	0.49	0.26	0.01	0.22
PYF-TT-*o*	1.39	0.869	0.52	0.26	0.02	0.24
PYF-DT-*o*	1.40	0.899	0.50	0.27	0.01	0.22

The energy losses of the all-PSCs were evaluated based on the equation below:

$$E_{loss} = E_g - qV_{OC} = \Delta E_1 + \Delta E_2 + \Delta E_3$$

where q is the elementary charge, ΔE_1 is the radiative energy loss above the bandgap, ΔE_2 is the radiative energy loss below the bandgap, and ΔE_3 is the non-radiative energy loss.

Table S4. Charge carrier mobilities of PM6:polymer acceptor blends.

Donor:acceptor	μ_e (10^{-4} cm^{2}V^{-1}s^{-1})	μ_h (10^{-4} cm^{2}V^{-1}s^{-1})	μ_h/μ_e
PM6:PYF-T-*o*	7.8	6.6	0.85
PM6:PYF-V-*o*	8.6	6.9	0.80
PM6:PYF-TT-*o*	7.2	6.5	0.90
PM6:PYF-DT-*o*	6.8	5.6	0.82

References

[1] H. Yu, M. Pan, R. Sun, I. Agunawela, J. Zhang, Y. Li, Z. Qi, H. Han, X. Zou, W. Zhou, S. Chen, J. Y. K. Lai, S. Luo, Z. Luo, D. Zhao, X. Lu, H. Ade, F. Huang, J. Min, H. Yan, *Angew. Chem. Int. Ed.* **2021**, 60, 10137-10146.

[2] P. Josse, C. Dalinot, Y. Jiang, S. Dabos-Seignon, J. Roncali, P. Blanchard, C. Cabanetos, *J. Mater. Chem. A.* **2016**, 4, 250.

[3] L. Ye, Y. Xiong, H. Yao, A. Gadisa, H. Zhang, S. Li, M. Ghasemi, N. Balar, A. Hunt, B. T. O'Connor, J. Hou, H. Ade, *Chem. Mater.* **2016**, 28, 7451-7458.

[4] Y. Xu, J. Yuan, S. Zhou, M. Seifrid, L. Ying, B. Li, F. Huang, G. C. Bazan, W. Ma, *Adv. Funct. Mater.* **2019**, 29, 1806747.

[5] Y. Guo, Y. Li, O. Awartani, J. Zhao, H. Han, H. Ade, D. Zhao, H. Yan, *Adv. Mater.* **2016**, 28, 8483-8489.

[6] B. Lin, L. Zhang, H. Zhao, X. Xu, K. Zhou, S. Zhang, L. Gou, B. Fan, L. Zhang, H. Yan, X. Gu, L. Ying, F. Huang, Y. Cao, W. Ma, *Nano Energy* **2019**, 59, 277-284.

[7] H. Yu, S. Luo, R. Sun, I. Angunawela, Z. Qi, Z. Peng, W. Zhou, H. Han, R. Wei, M. Pan, A. M. H. Cheung, D. Zhao, J. Zhang, H. Ade, J. Min, H. Yan, *Adv. Funct. Mater.* **2021**, 31, 2100791.

[8] B. Liu, H. Sun, J. -W. Lee, J. Yang, J. Wang, Y. Li, B. Li, M. Xu, Q. Liao, W. Zhang, D. Han, L. Niu, H. Meng, B. J. Kim, X. Guo, *Energy Environ. Sci.* **2021**, 14, 4499.
